# Vascular endothelial growth factor and risk of malignant brain tumor: A genetic correlation and two-sample Mendelian randomization study

**DOI:** 10.3389/fonc.2023.991825

**Published:** 2023-02-23

**Authors:** Qiaoyun Zhang, Guangheng Wu, Xiaoyu Zhang, Jie Zhang, Mengyang Jiang, Yiqiang Zhang, Lixiang Ding, Youxin Wang

**Affiliations:** ^1^ Beijing Key Laboratory of Clinical Epidemiology, School of Public Health, Capital Medical University, Beijing, China; ^2^ Department of Anesthesiology, Beijing Sanbo Brain Hospital, Capital Medical University, Beijing, China; ^3^ Department of Spine, Beijing Shijitan Hospital, Capital Medical University, Beijing, China

**Keywords:** vascular endothelial growth factor, malignant brain tumor, Mendelian randomization, causal inference, genetic correlation

## Abstract

**Objective:**

The relationship between vascular endothelial growth factor (VEGF) and the risk of malignant brain tumors has always been a concern in the medical field. However, the causal inferences from published observational studies on this issue may be affected by confounders, coinheritability and reverse causality. We aimed to investigate the causal relationship between VEGF and different types of malignant brain tumors.

**Methods:**

Using publicly available summary data from genome-wide association studies (GWAS) of VEGF (n=16,112) and different types of malignant brain tumors (n=174,097-174,646), we adopted a standard two-sample bidirectional Mendelian randomization (MR) to estimate potential causal associations of circulating VEGF levels and the risk of malignant brain tumors. Inverse variance weighted (IVW) was used as the primary analysis method to estimate causality. MR-Egger regression, weighted median (WM), penalty weighted median (PWM), MR robust adjusted profile score (MR.RAPS) and causal analysis using summary effect estimates (CAUSE) methods were used in sensitivity analyses to verify the robustness of the findings. Meanwhile, we applied the MR pleiotropy residual sum and outlier (MR-PRESSO) test and PhenoScanner tool to identify and remove potential horizontal pleiotropic single nucleotide polymorphisms (SNPs). Additionally, linkage disequilibrium score regression (LDSC) analysis was conducted to assess the coinheritability of exposure and outcome.

**Results:**

A total of 6 (VEGF), 12 (malignant brain tumor), 13 (brain glioblastoma) and 12 (malignant neoplasm of meninges) SNPs were identified as valid instrumental variables. No evidence supported a causal relationship between circulating VEGF levels and the risk of malignant brain tumors (forwards: odds ratio (OR) = 1.277, 95% confidence interval (CI), 0.812~2.009; reversed: β = 0.005, 95% CI, -0.029~0.038), brain glioblastoma (forwards: OR (95% CI) = 1.278(0.463~3.528); reversed: β = 0.010, 95% CI, -0.002~0.022) and malignant neoplasm of meninges (forwards: OR (95% CI) = 0.831(0.486~1.421); reversed: β = 0.010, 95% CI, -0.030~0.050) using the main IVW method. Outliers and pleiotropy bias were not detected by sensitivity analyses and pleiotropy-robust methods in any estimates. LDSC failed to identify genetic correlations between VEGF and different types of malignant brain tumors.

**Conclusions:**

Our findings reported no coinheritability and failed to provide evidence for causal associations between VEGF and the risk of different types of malignant brain tumors. However, certain subtypes of VEGF for which genetic predictors have not been identified may play a role and need to be further investigated.

## Introduction

Brain tumors, which account for more than 90% of central nervous system (CNS) tumors, are responsible for substantial cancer-related morbidity and mortality worldwide. The incidence rate and mortality rate of brain tumors significantly related to the human development index increased from 1990 to 2016 (1, 2) and may continue to increase with the development of society. Although malignant brain tumors (e.g., glioblastoma or malignant meninges) account for only approximately 20% of brain tumors, the one-year and five-year relative survival after diagnosis with a malignant brain tumor was low (56.6% and 32.1%, respectively) (3). Currently, ionizing radiation is the only well-recognized risk factor, while allergic and atopic diseases are protective factors (3, 4), but other genetic and environmental risk factors that can predict the risk of malignant brain tumors remain unclear (5).

Angiogenesis, one of the most typical pathological manifestations, is necessary for the growth and metastasis of invasive tumors (6–8). Vascular endothelial growth factor (VEGF) was identified as one of the most effective tumor angiogenesis factors (TAFs) and is known to affect various disease processes (cancers (9), cardiovascular diseases (10), etc.) and is pharmacologically modifiable (11). Given the important role of VEGF in tumor growth and invasion through promoting angiogenesis (12, 13), observational studies pointed to VEGF as a potential biomarker for brain tumors (14, 15). At the same time, the circulating VEGF level of patients with a variety of cancers (e.g., uterine cancer, ovarian cancer and lung cancer) was significantly higher than that of healthy controls (16). However, findings on VEGF levels in patients with brain tumors are inconsistent (15, 17). Therefore, we conducted a conventional two-sample Mendelian randomization study to analyze the causal relationship between VEGF and the risk of malignant brain tumors.

The design of the Mendelian randomization (MR) study follows Mendel’s law of inheritance, which is similar to randomized controlled trials and may provide more robust evidence for causal estimation between VEGF and malignant brain tumor risk. Genetic variants robustly related to VEGF and malignant brain tumors were selected as instrumental variables (IVs). IVs are less likely to be influenced by confounders or reverse causality due to the random assignment of parents to offspring at conception (18, 19). Linkage disequilibrium score (LDSC) regression was performed to explore the coheritability of VEGF with malignant brain tumors by assessing the genetic correlation (20). This study focused on VEGF, the most prominent mediator of tumor-associated angiogenesis (21), the correlation of which with the risk of malignant brain tumors is not yet well defined (15). Here, we applied univariable MR (UVMR) and bidirectional MR methods to detect the causal association of VEGF with the risk of malignant brain tumors using summary GWAS data from European populations.

## Materials and methods

**Figure 1:**
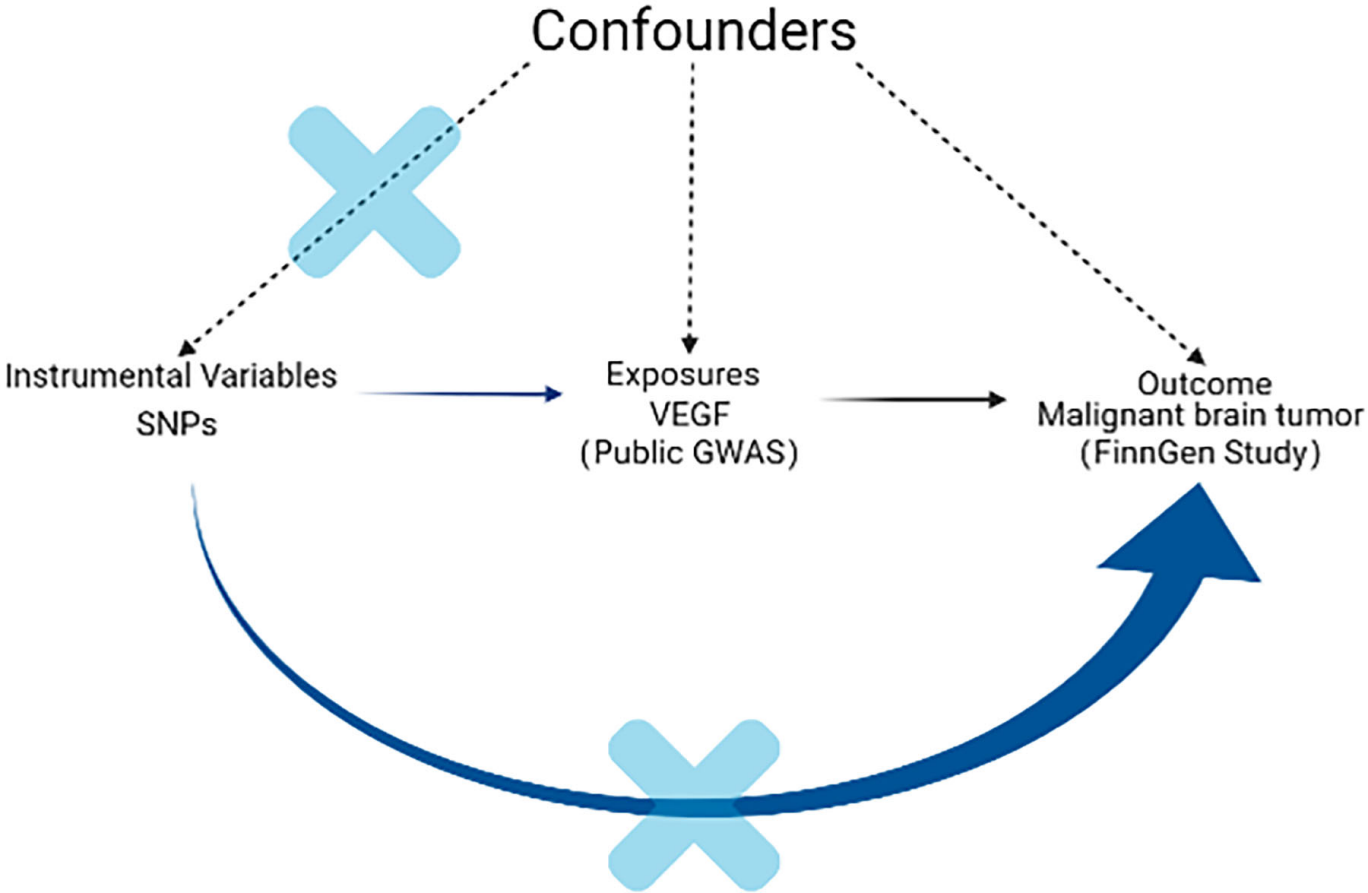
Schematic diagram of the MR design. The instrumental SNP was based on three key assumptions as follows: (1) the SNPs significantly related to exposure (VEGF) were selected as the instrumental variable (*P* < 5 × 10^-8^). (2) The SNPs have no association with confounders. Since genes are randomly assigned to the next generation of individuals following gametes during meiosis, there is little correlation between SNPs and confounding factors. (3) The SNPs should only be linked with outcome *via* exposure. Through sensitivity analysis such as MR-PRESSO, we excluded horizontal pleiotropy and restrictive assumptions to ensure that SNP affect the outcome (malignant brain tumor) only through exposure (VEGF). VEGF: vascular endothelial growth factor; SNP: single-nucleotide polymorphism; MR: Mendelian randomization; MR-PRESSO: pleiotropy residual sum and outlier.

A schematic diagram of the MR design and three major assumptions of MR are shown in **Figure 1**. All statistical analyses in this study were based on available summary data; therefore, no ethical approval was required.

### GWAS of VEGF

The summary data of VEGF were derived from the largest published GWAS meta-analysis studies (including ten studies) based on 16,112 individuals of European ancestry. The units of VEGF level were pg/ml and were natural log-transformed; other details are provided elsewhere (10).

### GWAS of malignant brain tumors

We used summary data from a GWAS that was made public by the FinnGen consortium (Release 5, https://www.finngen.fi/en), including 464 cases and 174,006 controls for malignant brain tumors (all cancers excluded), 91 cases and 174,006 controls for brain glioblastoma (all cancers excluded) and 640 cases and 174,006 controls for malignant neoplasm of meninges (all cancers excluded).

All GWAS summary data on exposure and outcomes were based on the European population.

### Genetic instrumental variables for VEGF

Single nucleotide polymorphisms (SNPs) significantly (*P* < 5×10^-8^) related to VEGF levels were selected as instrumental variables (IVs). Since only 3 SNPs were retained after a harmonizing step at r^2^ < 0.001, all independent variants (r^2^ < 0.01) were retained based on European ancestry reference data from the 1000 Genomes Project. In addition, the Phenoscanner (22, 23) (http://www.phenoscanner.medschl.cam.ac.uk/) search was used to check or detect whether any of these selected SNPs were strongly related to other diseases or phenotypes other than VEGF, so as to prevent a possible effect of the genetic variants on the outcome through confounding factors, known as horizontal pleiotropy. We looked up each SNP and their proxies (r^2^ > 0.80) to check any previous associations (*P <*5×10^-8^) with 3 potential confounders selected based on previously published studies: ulcerative colitis (24–26), interleukin (IL) levels (27–29) and hemoglobin concentration (30). Three SNPs were detected and eliminated for being associated with potential confounders (rs6920532: colitis ulcerative, rs6921438: interleukin (IL) (IL-12p70, IL-10, IL-13, IL-7 and IL-5) levels, and rs34881325: hemoglobin concentration).

### Genetic instrumental variables for malignant brain tumors

SNPs that were significantly (*P* < 1×10^-5^) associated with the different types of malignant brain tumors were selected as IVs. Since the number of independent SNPs of malignant brain tumors was limited, we selected eligible SNPs by relaxing the GWAS *P* threshold to 1×10^-5^ when it was treated as exposure. Independent variables from each other were retained based on European ancestry reference data from the 1000 Genomes Project (linkage disequilibrium (LD), r^2^ < 0.001). As above, we also used the PhenoScanner tool to manually remove SNPs and their proxies (r^2^ > 0.80) that were significantly (*P <*5×10^-8^) associated with potential confounders of the VEGF-malignant relationship based on published studies: white cell (31, 32). One SNP (rs147958197) for malignant brain tumors was associated with monocyte count, monocyte percentage of white cells or granulocyte percentage of myeloid white cells.

### MR analysis

Forwards MR analyses were conducted to assess the causal effect of the circulating VEGF level on malignant brain tumor risk. Then, reverse MR was performed using genetic variants with malignant brain tumors to investigate its causal effect on VEGF. The SNP effects (i.e., beta) and the corresponding standard errors (SE) were obtained from the GWAS-VEGF and GWAS-malignant brain tumor (33). Next, palindromic SNPs were removed by harmonizing VEGF and malignant brain tumor data (34).

Inverse variance weighted (IVW) MR was performed as the main method, which was actually a single variable weighted linear regression of outcome (SNPs) effects on exposure (SNPs) effects, and the intercept was constrained to zero (35). The results may be imprecise if IVs exhibit horizontal pleiotropy, meaning that IVs may affect outcomes *via* pathways other than the exposures (36). Therefore, we supplementarily applied several MR methods based on different IV assumptions, including MR-Egger regression, weighted median (WM), penalty weighted median (PWM), robust adjusted profile score (MR.RAPS) and causal analysis using summary effect estimates (CAUSE) as sensitivity analyses to verify the robustness of our main IVW estimate (36). The MR-Egger regression (the intercept is not constrained to zero (36, 37)) gives consistent estimates with the IVW method if all IVs are invalid, while WM and PWM methods require more than half of the IVs to be valid (38). For efficiency, WM estimates are generally as accurate as IVW estimates, both are more accurate than MR-Egger estimates, and MR-Egger regression estimates are especially imprecise if the IVs are all similarly associated with the exposure (38). The MR.RAPS method is robust to both systematic and idiosyncratic pleiotropy and can make our results more reliable when many weak IVs exist (39). Horizontal pleiotropy may be uncorrelated (IVs affect exposure and outcome *via* independent pathways) and correlated (IVs affect exposure and outcome through shared factors) with a shared factor, but both do not violate the major MR assumptions (40). CAUSE analysis, a recent method that accounts for correlated or uncorrelated horizontal pleiotropy effects, was conducted, which includes more IVs by LD pruning (LD r^2^ < 0.1) with its built-in function based on precomputed LD estimates (40).

Horizontal pleiotropy was evaluated by the intercept test of MR-Egger (the intercept *p*-value < 0.05 implied the presence of horizontal pleiotropy) (41) and the MR pleiotropy residual sum and outlier (MR-PRESSO) test (potential outlier SNPs that violated the IV assumptions could be detected) (42). As a sensitivity analysis, MR-PRESSO contains three components, known as the global test (detection of horizontal pleiotropy), outlier test (correction by removal of offending SNPs that are due to horizontal pleiotropy) and distortion test (testing of significant differences in the causal estimates before and after outlier removal). The global test is the key evaluation component of horizontal pleiotropy, and a *P* value greater than 0.05 indicates no pleiotropic effects (42). Additionally, heterogeneity was estimated by the Cochran *Q* test and *I^2^* statistics in the IVW and MR-Egger methods (Cochran Q_*P* value < 0.05 or *I^2^* statistics > 25% indicated the presence of heterogeneity) (43–45), which could help to evaluate horizontal pleiotropy. The leave-one-out and funnel plots were used to assess potential outliers or asymmetry visually (36).

Odds ratios (ORs) and the corresponding 95% confidence intervals (CIs) of malignant brain tumors correspond to malignant brain tumor risk per SD increase in log odds of the circulating VEGF level; alternatively, β and the corresponding 95% CI of VEGF represent the reverse association. Bonferroni correction was performed to account for three outcomes, and *P* < 0.017 (0.05/3) was defined as a statistically significant difference. MR analysis was conducted by using the following R (version 4.0.3, https://cran.r-project.org/) packages: “TwoSampleMR”, “MR-PRESSO” and “CAUSE”.

### Variance explained by IVs and F_Statistic analyses

To estimate the variance explained for each SNP, we calculated R^2^ by the following formula: R^2^ = 2×MAF× (1-MAF) × Beta^2^. Then, we summed the R^2^ to calculate the overall R^2^ and F statistics for exposure (F_statistic = R^2^ × (N-2)/(1-R^2^)), where N means the number of individuals of the GWAS-exposure (46). The higher the R^2^ and F statistics are, the lower the risk of weak IVs bias (47).

### LDSC regression analysis

LDSC regression regressed χ^2^ statistics for one trait to calculate SNP-based heritability (h^2^) or two traits to estimate SNP-based coheritability (http://ldsc.broadinstitute.org/ldhub/, LD score tool, version 1.0.1). Cross-trait LDSC regression was conducted to assess the genetic correlations between VEGF and malignant brain tumors by the regression slope using GWAS summary data (20). If the heritability z-score is small (i.e., < 4), the genetic correlation estimates are generally too noisy to assess (48). Likewise, the results are probably not suitable for LDSC regression with a small chi-square (e.g., < 1.020) (49).

## Results

Detailed information on the characteristics of the SNPs used for each trait is shown in the **Supplementary material** (**Supplementary Tables 1, 2**). Brief information on the GWAS data is listed in **Table 1**.

**Table 1:** Brief information on exposures used in the MR analyses.

Exposure		Outcome		nIVs	R^2^	F_statistic
Trait	Sample	Trait	Sample			
VEGF (r^2^<0.01)	16,112	malignant brain tumor	174,470	6	0.035	7643.548
malignant brain tumor (r^2^<0.001)	174,470	VEGF	16,112	12	0.643	118736.841
Brain glioblastoma (r^2^<0.001)	174,097	VEGF	16,112	13	3.919	996291.187
malignant neoplasm of meninges (r^2^<0.001)	174,646	VEGF	16,112	12	0.451	81822.266

VEGF, vascular endothelial growth factor; MR, Mendelian randomization; R^2^, variance for SNPs; nIVs, number of instrumental variables.

### Causal effect of VEGF on the risk of malignant brain tumors *via* forwards MR

In the forwards MR analyses, a total of 11 SNPs were screened, and the F-statistics ranged from 291 to 29,730. Nine independent SNPs (rs7030781 and rs10761731 were excluded for being palindromic structures) were retained after harmonizing SNP-exposure and SNP-outcomes. Then, three SNPs were removed for being associated with potential confounders *via* the PhenoScanner tool. Finally, 6 SNPs were selected as the IVs for VEGF, and these IVs could explain 3.5% of the variance in VEGF levels.

**Figure 2:**
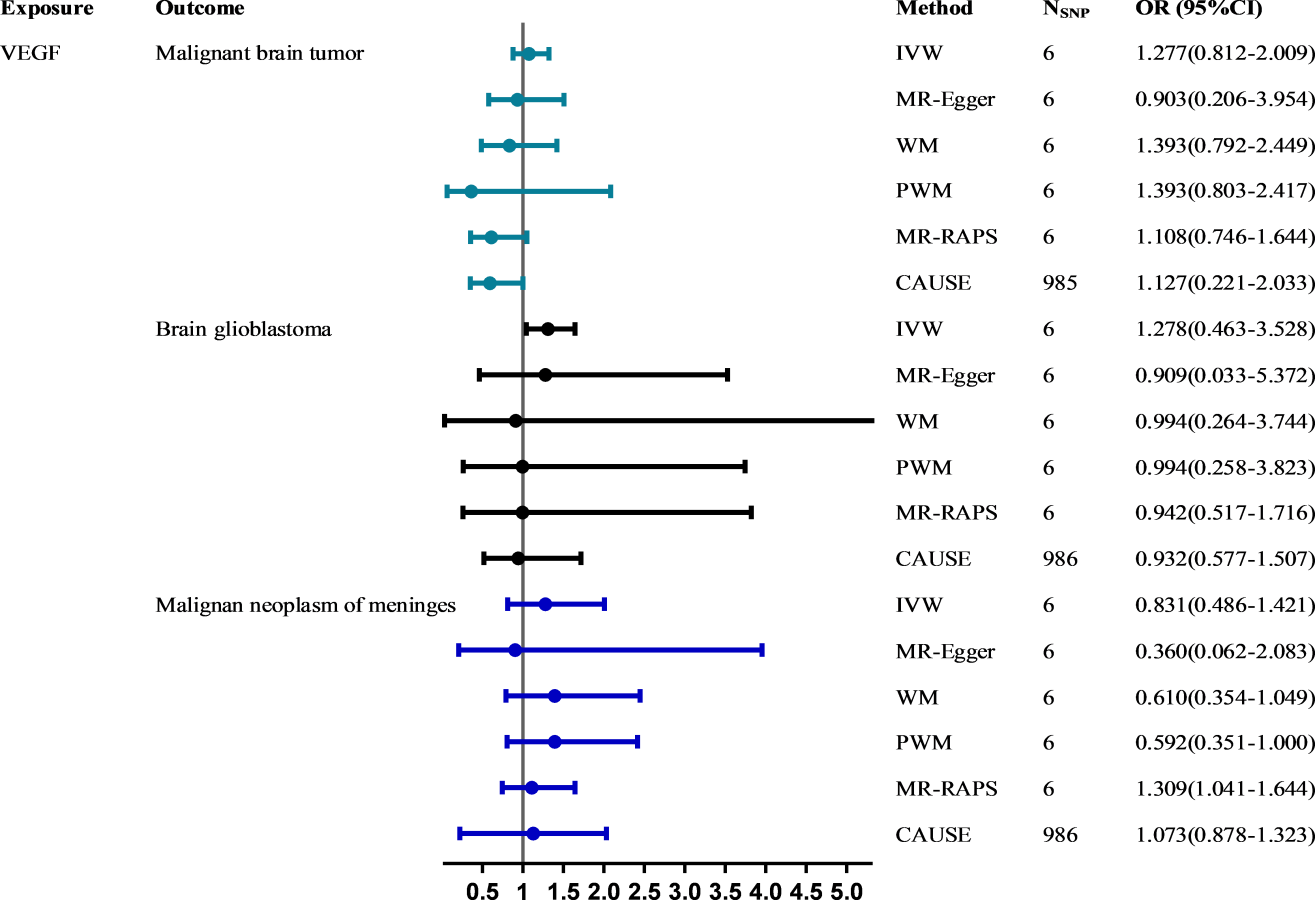
The causal effect of VEGF on the risk of malignant brain tumors estimated using six MR methods. The causal effect of VEGF on malignant brain tumor risk was expressed as the OR per unit. Error bars represent the 95% CIs of the estimates. CAUSE recruited independent instrumental SNPs with GWAS *p* values < 1×10^-3^. SNP: single-nucleotide polymorphism; VEGF: vascular endothelial growth factor; IVW: inverse-variance weighted; WM: weighted median; PWM: penalty weighted median; MR.RAPS: robust adjusted profile score; CAUSE: causal analysis using summary effect estimates; OR: odds ratio; SD: standard deviation; CIs: confidence intervals; MR: Mendelian randomization.

**Figure 2** shows the MR estimates for VEGF on different types of malignant brain tumor risk using different methods. The main MR methods indicated that VEGF was not associated with malignant brain tumors (IVW: OR = 1.277, 95% CI, 0.812~2.009, *P* = 0.289) without heterogeneity (*Q*_pval = 0.572, *I^2^* = 0.000) and horizontal pleiotropy (intercept = 0.054, *P* = 0.654). The causal estimates for VEGF on the risk of brain glioblastoma (IVW: OR = 1.278, 95% CI, 0.463~3.528, *P* = 0.636) and malignant neoplasm of meninges (IVW: OR = 0.831, 95% CI, 0.486~1.421, *P* = 0.499) were basically consistent with the above findings. CAUSE analyses indicated that the sharing model was better than the causal model, and other robust analysis methods provided consistent results. The leave-one-out and MR-PRESSO analyses did not detect any potential outlier SNPs (all global tests *P* > 0.05) (**Supplementary Figure 1**). There was no directional pleiotropy, as the funnel plots detected no evidence of asymmetry (**Supplementary Figure 2**). Please see **Supplementary Tables 3, 4** in the **supplementary material** for detailed results.

### Causal effect of malignant brain tumors on VEGF *via* reverse MR

In the reverse MR analyses, a total of 17 SNPs were selected for malignant brain tumors, and the F statistics ranged from 8,153 to 13,940. Thirteen (rs11207597, rs148088011, rs55737523, rs73161249 were excluded for being palindromic structures) independent SNPs were retained after harmonizing SNP-exposure and SNP-outcomes. Then, one SNP (rs147958197) was removed for being associated with monocyte count, monocyte percentage of white cells or granulocyte percentage of myeloid white cells *via* the PhenoScanner tool. Finally, 12 SNPs were selected as IVs and those SNPs could explain up to 64.3% of the variance in malignant brain tumors. The number of IVs selected for brain glioblastoma and malignant neoplasm of meninges (strong correlation with the exposure (*P* < 1×10^-5^), but not with the corresponding outcome (*P* > 5 × 10^-8^)) through the same steps was 13 and 12, respectively (**Supplementary Tables 2-2, 2-3**).

**Figure 3:**
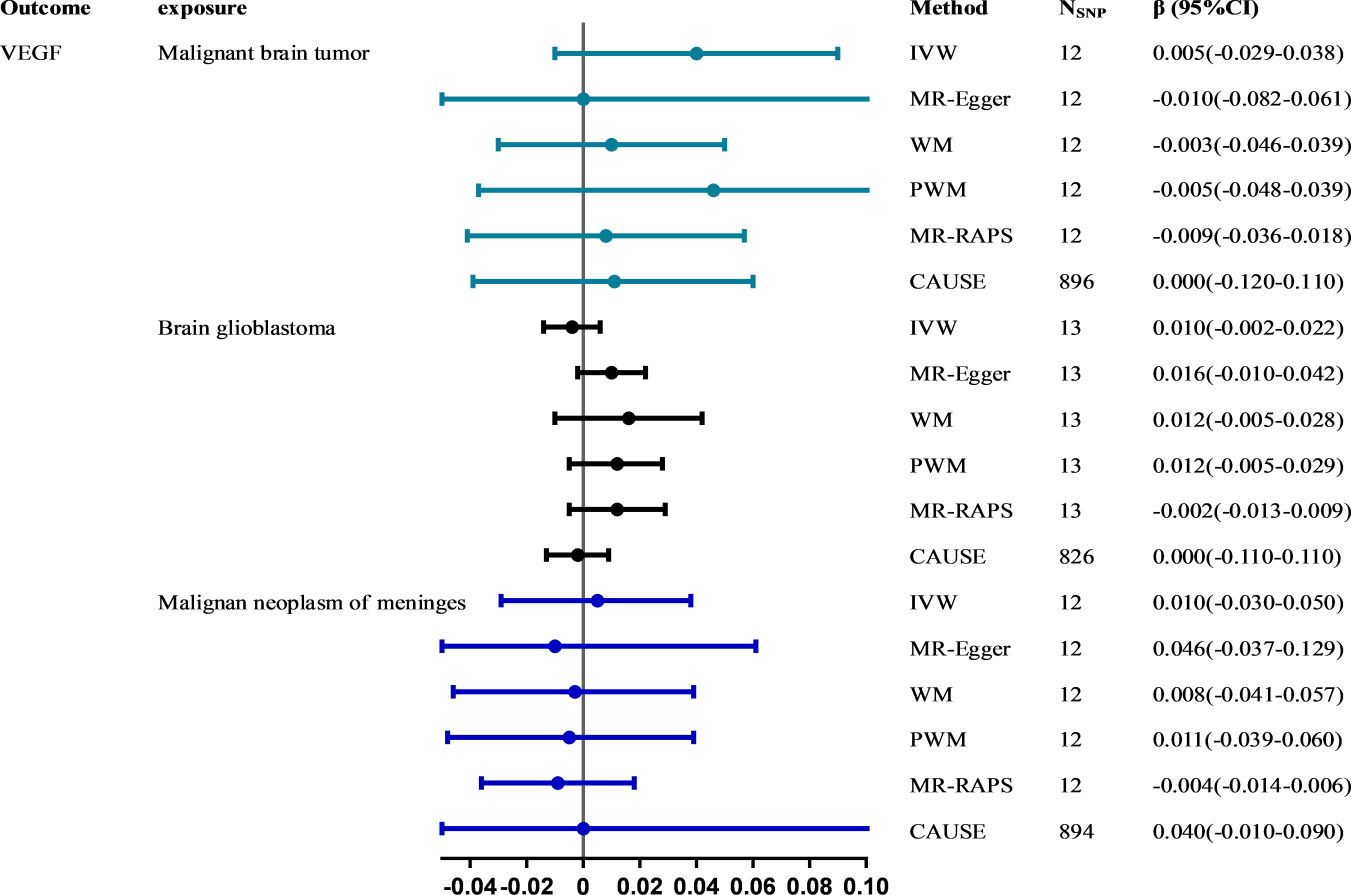
The causal effect of malignant brain tumors on VEGF estimated using six MR methods. The causal effect from malignant brain tumors to VEGF was expressed as β. Error bars represent the 95% CIs of the estimates. CAUSE recruited independent instrumental SNPs with GWAS *p* values < 1×10^-3^. SNP: single-nucleotide polymorphism; VEGF: vascular endothelial growth factor; IVW: inverse-variance weighted; WM: weighted median; PWM: penalty weighted median; MR.RAPS: robust adjusted profile score; CAUSE: causal analysis using summary effect estimates; CIs: confidence intervals; MR: Mendelian randomization.

All six methods in reverse MR analyses consistently suggested no significant association of genetically instrumented malignant brain tumors with VEGF (IVW: β = 0.005; 95% CI, -0.029~0.038; *P* = 0.790) (**Figure 3** and **Supplementary Table 5**). There was no evidence of heterogeneity between IV estimates with IVW methods from individual SNPs (*Q*_pval = 0.285, *I^2^* = 0.162) and no pleiotropy effect (intercept = 0.009, *P* = 0.652). CAUSE analyses indicated that the sharing model was better than the causal model (**Supplementary Table 4**), and other robust analysis methods provided consistent results. No pleiotropic outliers were detected according to the leave-one-out and MR-PRESSO tests (all global tests *P* > 0.05) (**Supplementary Figure 3**). No directional pleiotropy was found, as the funnel plots showed no evidence of asymmetry (**Supplementary Figure 4**). The causal estimates for brain glioblastoma (IVW: β = 0.010, 95% CI, -0.002~0.022, *P* = 0.108) and malignant neoplasm of meninges (IVW: β= 0.010, 95% CI, -0.030~0.050, *P* = 0.618) on VEGF were similar to those described above.

### LDSC regression analyses

The total heritability of VEGF (3.5%) and the different types of malignant brain tumors (0.2-0.5%) was relatively small (**Table 2**). Therefore, the genetic correlation between VEGF and different types of malignant brain tumors was not obtained (48) (**Table 3**).

**Table 2:** The heritability of VEGF and different types of malignant brain tumors.

Traits	h^2^ (SE)	Mean Chi^2^	Intercept	Ratio
VEGF	0.035 (0.029)	1.020	1.009 (0.010)	0.454 (0.505)
malignant brain tumor	0.002 (0.003)	1.013	1.007 (0.007)	0.562 (0.580)
Brain glioblastoma	-0.003(0.003)	0.989	1.001(0.007)	NA
Malignant neoplasm of meninges	0.005(0.003)	1.008	0.992(0.007)	<0

VEGF, vascular endothelial growth factor; SE, standard error. NA, not available.

**Table 3:** Genetic correlation between VEGF and different types of malignant brain tumors.

P1 name	P2 name	rg	se	*P*	h^2^_obs (se)	h^2^_int (se)
VEGF	malignant brain tumor	–	–	–	0.002(0.003)	1.007(0.007)
VEGF	Brain glioblastoma	–	–	–	-0.004(0.003)	1.003(0.007)
VEGF	Malignant neoplasm of meninges	–	–	–	0.005(0.003)	0.991(0.007)

VEGF, vascular endothelial growth factor.

## Discussion

VEGF plays an important role in impacting various physiological processes of cancer and disease, but its function in the formation and progression of malignant brain tumors remains unclear, and anti-VEGF therapies have had little clinical efficacy, highlighting the need to explore VEGF-independent mechanisms of angiogenesis (50). Although VEGF might be a potential biomarker or risk factor, as shown in observational studies, our study failed to obtain convincing evidence that VEGF increases the risk of malignant brain tumors, brain glioblastoma and malignant neoplasm of meninges using the strong IVs from GWAS summary data, and vice versa.

The pathogenic effects of VEGF are mainly due to its effect on vascular permeability and neovascularization (51). In addition, angiogenesis is a prominent feature of aggressive malignancies (21), including brain tumors, and the level of circulating VEGF is positively associated with tumor aggressiveness, making VEGF a promising therapeutic target for high-grade malignant brain tumors (52, 53). Unexpectedly, we found no causal relationship between VEGF and malignant brain tumors, and the genetic correlation analysis also yielded consistent results (no coinheritance existed). Although the exact mechanisms remain to be elucidated, the variant subtypes of VEGF and different sites of origin may be considered. The VEGF protein family (i.e., VEGF-A, B, C) is involved in a variety of pathophysiological processes (54). Although VEGF-A is the main genetic locus-determining circulating VEGF levels (55), blocking tumor derived VEGF-A alone cannot inhibit tumor growth sufficiently. Host-derived (e.g., myeloid cells) VEGF-A may compensate for the lack of tumor-derived VEGF-A (56), which is consistent with the importance of VEGF-A derived from myeloid cells in the early phases of glioma growth (57). In addition, vasorin (VASN), a hypoxia inducible factor-1 (HIF-1) protein induced by hypoxia, may be another consideration (58). Observational studies have found that angiogenic factors (CCR2, VEGF-A, etc.) are overexpressed in glioma cells with high levels of VASN (59). Namely, the correlation between brain tumors and VEGF may be due to the upregulation of the VEGF concentration by VASN. Although the relevant mechanism remains to be addressed, the promoting effect of VASN on angiogenesis may be mainly attributed to the concomitant upregulation of angiogenic factor concentrations by VASN. Furthermore, previous studies also pointed out that the levels of plasminogen activator inhibitor-1 (PAI-1) and tissue-type plasminogen activator (TPA) in patients with brain tumors are increased (15). These molecules secreted by tumor cells may lead to the elevation of VEGF levels (60). Therefore, VEGF is a factor related to increased TF expression or activity in malignant glioma (61). In summary, the correlation between VEGF and brain tumors might not indicate that there is a causal relationship. The potential mechanism of different VEGF subtypes as well as additional functional and association studies in different ethnic populations await further exploration (15).

Although the design of MR studies is less susceptible to confounding factors and reverse causality, limitations exist. First, our study focused on the circulating level of VEGF, and the conclusion cannot be generalized to the function of the intracellular level of VEGF on malignant brain tumor risk. Second, the summary GWAS data used in this study were derived from the European population, so our conclusions may not generalize to other ethnic populations. Third, the VEGF family includes multiple subtypes (VEGF-A, VEGF-D, etc.) (54), and limited by current knowledge and the inability to obtain both individual-level and summary data for GWAS of VEGF-subtypes and risk factors for malignant brain tumors to assess potential genetic correlations, we cannot explore other exposures and cannot rule out the possibility of pleiotropic effects. Nonetheless, we performed MR-Egger regression, MR.RAPS and CAUSE analyses, which were more robust to invalid SNPs and considered the correlated and uncorrelated pleiotropy effects; fourth, the difference in the sample size of GWAS-VEGF and GWAS-malignant brain tumor may lead to unstable statistical results, and the identified SNPs may exhibit potential weak instrument bias, but this is less likely because the F statistics for each SNP used was significantly higher than ten. However, the small sample size of VEGF could be one of the important reasons for the failure of gene association analysis (LDSC). Therefore, data from larger samples need to be discovered and verified.

In conclusion, our findings reported no coheritability between VEGF and malignant brain tumors. This study also failed to provide evidence for the causal association between VEGF and the risk of malignant brain tumors. However, certain subtypes of VEGF for which genetic predictors have not been identified may play a role and need to be further investigated. The identification of patients at higher risk of malignant brain tumors may lead to a more targeted preventive treatment of those individuals. Additionally, MR studies using individual-level statistics may be beneficial to elucidate the potential nonlinear relationship between VEGF levels and malignant brain tumor risk.

## Data availability statement

Publicly available datasets were analyzed in this study. This data can be found here: The FinnGen consortium (Release 5, https://www.finngen.fi/en) https://doi.org/10.1371/journal.pgen.1005874 or https://grasp.nhlbi.nih.gov/FullResults.aspx.

## Author contributions

YW, QZ, and LD conceived this study. QZ and GW performed the data analyses and were the comajor contributors in writing this manuscript. XZ, JZ, MJ, and YZ contributed to methodological guidance and manuscript revision. YW and LD oversaw the implementation of the statistical and analytical methods, and revised and finalized this manuscript. All authors contributed to the article and approved the submitted version.
