# Supplementary material for: Vascular endothelial growth factor and risk of malignant brain tumor: A genetic correlation and two-sample Mendelian randomization study
Source: Front Oncol. 2023 Feb 23;13:991825. doi: 10.3389/fonc.2023.991825 (PMC9995857; doi:10.3389/fonc.2023.991825)
Supplement: Supplementary file 1 [file DataSheet_1.docx]

Supplementary Material

1. **Supplementary Tables**

**Supplementary Table 1** SNPs for VEGF in the forwards MR analyses: Harmonized data (r^2^<0.01)

|  |  |  |  |  |  |  |  |  |  |  | Outcome: malignant brain tumor | | |
| --- | --- | --- | --- | --- | --- | --- | --- | --- | --- | --- | --- | --- | --- |
| SNP | CHR | POS | OA | EA | MAF | Beta | SE | *P* | R^2^ | F_statistic | Beta | SE | *P* |
| rs9381249 | 6 | 43734798 | T | C | 0.0109 | 0.2482 | 0.0397 | 3.09E-10 | 0.001328312 | 291.0080349 | -0.016 | 0.1648 | 0.9227 |
| rs6920532^$^ | 6 | 43793430 | T | C | 0.8936 | -0.1803 | 0.0267 | 8.68E-12 | 0.006181676 | 1360.90154 | 0.0789 | 0.1086 | 0.4672 |
| rs67798973 | 6 | 43882777 | A | G | 0.4433 | -0.1389 | 0.0175 | 1.29E-15 | 0.009522554 | 2103.469975 | 0.0761 | 0.0677 | 0.2609 |
| rs6921438^$^ | 6 | 43925607 | A | G | 0.4672 | 0.49 | 0.0175 | 2.09E-171 | 0.119533382 | 29703.23692 | -0.0363 | 0.0667 | 0.5866 |
| rs9472183 | 6 | 43940202 | A | G | 0.5109 | 0.1282 | 0.017 | 5.19E-14 | 0.008213715 | 1811.961565 | 0.0956 | 0.0666 | 0.1513 |
| rs74675876 | 6 | 43963995 | T | C | 0.0298 | 0.2822 | 0.0366 | 7.62E-15 | 0.004604914 | 1012.170149 | 0.0889 | 0.1496 | 0.5526 |
| rs4507572 | 6 | 44135095 | T | C | 0.4702 | -0.1007 | 0.0171 | 3.34E-09 | 0.005052235 | 1110.991415 | -0.0156 | 0.0666 | 0.8153 |
| rs41282660 | 6 | 44197006 | A | G | 0.8678 | 0.1613 | 0.0263 | 1.33E-09 | 0.005969663 | 1313.946435 | -0.1088 | 0.0983 | 0.2684 |
| rs34881325^$^ | 9 | 2622134 | T | C | 0.5736 | 0.1082 | 0.0189 | 1.04E-08 | 0.005726785 | 1260.180004 | -0.0424 | 0.07 | 0.545 |
| rs7030781^#^ | 9 | 2686273 | A | T | 0.5815 | -0.1368 | 0.0173 | 2.57E-15 | 0.009108511 | 2011.169813 | -0.0217 | 0.0680 | 0.7496 |
| rs10761731^#^ | 10 | 65027610 | A | T | 0.5676 | 0.1187 | 0.0174 | 1.01E-11 | 0.006916072 | 1523.705449 | 0.0782 | 0.0685 | 0.2538 |

SNP: single-nucleotide polymorphism; CHR: chromosome; POS: position; OA: other_allele; EA: effect_allele; MAF: minor allele frequency; SE: standard error; MR: Mendelian randomization; R^2^: variance for each SNP, R^2^ = 2×MAF× (1-MAF) × Beta^2^; F_statistic = R^2^ × (N-2) / (1-R^2^), N: the number of individuals in the exposure GWAS.

^$^: SNPs were removed via the PhenoScanner tool.

^#^: SNPs were excluded after performing harmonizing procedure.

**Supplementary Table 2-1** SNPs for malignant brain tumor in the reverse MR analyses: Harmonized data (r^2^ < 0.001)

|  |  |  |  |  |  |  |  |  |  |  | Outcome: VEGF | | |
| --- | --- | --- | --- | --- | --- | --- | --- | --- | --- | --- | --- | --- | --- |
| SNP | CHR | POS | OA | EA | MAF | Beta | SE | *P* | R^2^ | F_statistic | Beta | SE | *P* |
| rs11207597^#^ | 1 | 60396109 | G | C | 0.5053 | -0.3219 | 0.0669 | 1.49E-06 | 0.0518039 | 9531.928 | 0.0025 | 0.0169 | 0.8876 |
| rs4658437 | 1 | 245148860 | A | G | 0.3611 | 0.3136 | 0.0692 | 5.85E-06 | 0.04537770 | 8293.287 | 0.0071 | 0.0176 | 0.6790 |
| rs148088011^#^ | 2 | 171465648 | T | A | 0.02151 | 1.3027 | 0.2580 | 4.44E-07 | 0.071436 | 13422.07 | 0.0956 | 0.0572 | 0.0890 |
| rs77671862 | 2 | 196203358 | C | A | 0.03982 | 0.8559 | 0.1905 | 7.06E-06 | 0.05601830 | 10353.38 | -0.0514 | 0.0464 | 0.2665 |
| rs55737523^#^ | 2 | 240048413 | C | G | 0.3203 | 0.3448 | 0.0719 | 1.65E-06 | 0.051765 | 9524.423 | -0.0109 | 0.0185 | 0.5551 |
| rs10059500 | 5 | 136676469 | C | T | 0.01815 | 1.2741 | 0.2769 | 4.19E-06 | 0.05785739 | 10714.16 | 0.0407 | 0.0592 | 0.4261 |
| rs73161249^#^ | 7 | 83007604 | C | G | 0.0323 | 0.8974 | 0.1997 | 7.03E-06 | 0.050344 | 9248.999 | -0.0393 | 0.0493 | 0.4334 |
| rs4831407 | 8 | 13349267 | G | A | 0.9649 | -0.9536 | 0.1949 | 9.90E-07 | 0.06159591 | 11451.91 | 0.1040 | 0.0478 | 0.02841 |
| rs139696720 | 8 | 71184583 | C | T | 0.015 | 1.5824 | 0.3157 | 5.37E-07 | 0.07399290 | 13940.92 | 0.1368 | 0.0915 | 0.1483 |
| rs147958197^$^ | 8 | 129619149 | T | C | 0.09067 | 0.5656 | 0.1211 | 2.99E-06 | 0.05275139 | 9715.96 | 0.0145 | 0.0318 | 0.6664 |
| rs10966834 | 9 | 25283758 | A | G | 0.1499 | 0.436 | 0.0961 | 5.72E-06 | 0.04844786 | 8882.962 | 0.0152 | 0.0254 | 0.5499 |
| rs146130523 | 10 | 83750994 | T | C | 0.02525 | 1.0668 | 0.2311 | 3.93E-06 | 0.05602097 | 10353.9 | -0.009 | 0.0618 | 0.8679 |
| rs146375890 | 11 | 62041672 | A | G | 0.03286 | 0.9089 | 0.2052 | 9.47E-06 | 0.05250723 | 9668.497 | -0.0406 | 0.0461 | 0.3705 |
| rs1194817 | 11 | 87855074 | C | T | 0.7068 | -0.3282 | 0.0733 | 7.60E-06 | 0.04464447 | 8153.018 | 0.0227 | 0.0189 | 0.2247 |
| rs117011442 | 12 | 123895144 | G | A | 0.04487 | 0.757 | 0.1713 | 9.91E-06 | 0.04911796 | 9012.172 | -0.0081 | 0.0385 | 0.8436 |
| rs9542683 | 13 | 71398567 | A | C | 0.8079 | -0.3884 | 0.0859 | 6.20E-06 | 0.04682453 | 8570.701 | 0.0289 | 0.0223 | 0.1969 |
| rs1565017 | 15 | 59247323 | G | A | 0.4144 | 0.3236 | 0.0678 | 1.81E-06 | 0.05082388 | 9341.934 | 0.0042 | 0.0174 | 0.8168 |

SNP: single-nucleotide polymorphism; CHR: chromosome; POS: position; OA: other_allele; EA: effect_allele; MAF: minor allele frequency; SE: standard error; MR: Mendelian randomization; VEGF: vascular endothelial growth factor; R^2^: variance for each SNP, R^2^ = 2×MAF× (1-MAF) × Beta^2^; F_statistic = R^2^ × (N-2) / (1-R^2^), N: number of individuals in the exposure GWAS.

^$^: SNPs were removed via the PhenoScanner tool.

^#^: SNPs were excluded after performing harmonizing procedure.

**Supplementary Table 2-2** SNPs for brain glioblastoma in the reverse MR analyses: Harmonized data (r^2^ < 0.001)

|  |  |  |  |  |  |  |  |  |  |  | Outcome: VEGF | | |
| --- | --- | --- | --- | --- | --- | --- | --- | --- | --- | --- | --- | --- | --- |
| SNP | CHR | POS | OA | EA | MAF | Beta | SE | *P* | R^2^ | F_statistic | Beta | SE | *P* |
| rs529324 | 2 | 136920897 | G | A | 0.7475 | -0.8232 | 0.1793 | 4.41E-06 | 0.25580751 | 59843.13 | -0.0214 | 0.02 | 0.2845 |
| rs116691373 | 2 | 224870297 | C | A | 0.009556 | 4.451 | 1.0072 | 9.91E-06 | 0.37501726 | 104464.7 | -0.0568 | 0.0892 | 0.5159 |
| rs80329173 | 4 | 120041762 | T | C | 0.02673 | 2.7412 | 0.5822 | 2.50E-06 | 0.39097027 | 111761.3 | -0.0045 | 0.0522 | 0.9356 |
| rs389558 | 5 | 179218741 | C | T | 0.3589 | -0.7835 | 0.1599 | 9.51E-07 | 0.2824927 | 68543.65 | 0.008 | 0.018 | 0.6589 |
| rs7778345 | 7 | 54610132 | A | G | 0.325 | 0.7331 | 0.1627 | 6.59E-06 | 0.23579987 | 53718.36 | -0.0163 | 0.0183 | 0.3767 |
| rs10513202 | 9 | 113262679 | A | G | 0.07393 | 1.4367 | 0.3217 | 7.95E-06 | 0.28263549 | 68591.94 | 0.0168 | 0.0324 | 0.6112 |
| rs17145573 | 11 | 57201981 | G | A | 0.06005 | 1.7004 | 0.3644 | 3.06E-06 | 0.32639985 | 84359.52 | 0.0116 | 0.0356 | 0.7365 |
| rs11230859 | 11 | 62002500 | G | A | 0.6269 | -0.7237 | 0.1574 | 4.29E-06 | 0.24500258 | 56495.19 | 0.0254 | 0.0176 | 0.1508 |
| rs118107790 | 15 | 74188585 | G | A | 0.04473 | 1.9566 | 0.4273 | 4.66E-06 | 0.3271592 | 84651.2 | -0.0765 | 0.0432 | 0.09171 |
| rs78423355 | 17 | 81147027 | A | C | 0.02015 | 3.1245 | 0.6751 | 3.69E-06 | 0.38550117 | 109217.2 | 0.0403 | 0.0651 | 0.5098 |
| rs491806 | 19 | 55995166 | A | C | 0.8262 | -0.9449 | 0.2106 | 7.22E-06 | 0.256411 | 60032.99 | -0.0316 | 0.0219 | 0.1488 |
| rs73610138 | 20 | 4086737 | C | T | 0.08244 | 1.4012 | 0.309 | 5.76E-06 | 0.29703164 | 73561.95 | -0.0421 | 0.0299 | 0.1656 |
| rs11090513 | 22 | 27744502 | G | T | 0.2788 | 0.8035 | 0.1751 | 4.49E-06 | 0.25962723 | 61050.06 | 0.0041 | 0.0188 | 0.8202 |

SNP: single-nucleotide polymorphism; CHR: chromosome; POS: position; OA: other_allele; EA: effect_allele; MAF: minor allele frequency; SE: standard error; MR: Mendelian randomization; VEGF: vascular endothelial growth factor; R^2^: variance for each SNP, R^2^ = 2×MAF× (1-MAF) × Beta^2^; F_statistic = R^2^ × (N-2) / (1-R^2^), N: number of individuals in the exposure GWAS.

**Supplementary Table 2-3** SNPs for malignant neoplasm of meninges in the reverse MR analyses: Harmonized data (r^2^ < 0.001)

|  |  |  |  |  |  |  |  |  |  |  | Outcome: VEGF | | |
| --- | --- | --- | --- | --- | --- | --- | --- | --- | --- | --- | --- | --- | --- |
| SNP | CHR | POS | OA | EA | MAF | Beta | SE | *P* | R^2^ | F_statistic | Beta | SE | *P* |
| rs34504438 | 3 | 57186130 | A | C | 0.1668 | -0.3536 | 0.0768 | 4.15E-06 | 0.034753601 | 6288.040001 | 0.0491 | 0.0227 | 0.03063 |
| rs141103197 | 5 | 87573497 | T | C | 0.02459 | 0.8819 | 0.1966 | 7.26E-06 | 0.037309069 | 6768.324972 | 0.1079 | 0.0575 | 0.06112 |
| rs113346358 | 7 | 29789616 | C | T | 0.1055 | 0.4291 | 0.0938 | 4.81E-06 | 0.034752002 | 6287.740208 | 0.0355 | 0.0273 | 0.1908 |
| rs11793023 | 9 | 1.23E+08 | G | A | 0.05666 | 0.5785 | 0.1278 | 6.05E-06 | 0.035775157 | 6479.729781 | -0.0087 | 0.036 | 0.7821 |
| rs12146252 | 10 | 1.23E+08 | G | A | 0.01787 | 1.0691 | 0.2342 | 5.02E-06 | 0.040119932 | 7299.563324 | 0.0185 | 0.068 | 0.7685 |
| rs7944093 | 11 | 1.35E+08 | A | G | 0.7507 | 0.2955 | 0.0663 | 8.20E-06 | 0.032683884 | 5900.908858 | 0.0214 | 0.0213 | 0.3294 |
| rs6493184 | 15 | 30619313 | G | A | 0.5036 | 0.2541 | 0.0569 | 8.07E-06 | 0.032281731 | 5825.880204 | 0.0045 | 0.017 | 0.7921 |
| rs139268808 | 16 | 4491617 | G | A | 0.008994 | 1.552 | 0.345 | 6.86E-06 | 0.042938078 | 7835.31081 | -0.1128 | 0.0665 | 0.08658 |
| rs909342 | 20 | 63620172 | T | C | 0.5101 | 0.2823 | 0.0566 | 6.12E-07 | 0.039830386 | 7244.697006 | 0.0167 | 0.0168 | 0.303 |
| rs73203029 | 21 | 34987852 | C | T | 0.01681 | 1.1343 | 0.2394 | 2.15E-06 | 0.042529573 | 7757.456107 | 0.0137 | 0.0697 | 0.8343 |
| rs12168849 | 22 | 28806075 | A | G | 0.2011 | 0.3727 | 0.0718 | 2.07E-07 | 0.044632712 | 8158.993285 | -0.0083 | 0.0211 | 0.6956 |
| rs929070 | 22 | 40280997 | T | C | 0.7843 | -0.3127 | 0.0692 | 6.15E-06 | 0.033084008 | 5975.621001 | -0.0039 | 0.0208 | 0.8575 |

SNP: single-nucleotide polymorphism; CHR: chromosome; POS: position; OA: other_allele; EA: effect_allele; MAF: minor allele frequency; SE: standard error; MR: Mendelian randomization; VEGF: vascular endothelial growth factor; R^2^: variance for each SNP, R^2^ = 2×MAF× (1-MAF) × Beta^2^; F_statistic = R^2^ × (N-2) / (1-R^2^), N: number of individuals in the exposure GWAS.

**Supplementary Table 3** Causal associations of VEGF with the risk of different types of malignant brain tumors by forwards MR analyses

| Exposure | Outcome | Methods | nSNPs | OR (95%CI) | Beta (SE) | *P* | Q_pval(*I^2^*) | Intercept(*P*) | RSSobs | Global *P* |
| --- | --- | --- | --- | --- | --- | --- | --- | --- | --- | --- |
| VEGF | Malignant brain tumor | IVW | 6 | 1.277(0.812~2.009) | 0.245(0.231) | 0.289 | 0.572(0.000) |  |  |  |
|  |  | MR-Egger | 6 | 0.903(0.206~3.954) | -0.102(0.754) | 0.898 |  | 0.054(0.654) |  |  |
|  |  | WM | 6 | 1.393(0.792~2.449) | 0.332(0.288) | 0.249 |  |  |  |  |
|  |  | PWM | 6 | 1.393(0.803~2.417) | 0.332(0.281) | 0.238 |  |  |  |  |
|  |  | MR.RAPS | 6 | 1.108(0.746~1.644) | 0.102(0.202) | 0.612 |  |  |  |  |
|  |  | CAUSE | 985 | 1.127(0.221~2.033) | 0.120(0.410) | 0.770 |  |  |  |  |
|  |  | MR-PRESSO | 6 | - | 0.100(0.184) | 0.602 |  |  | 8.170 | 0.525 |
| VEGF | Brain glioblastoma | IVW | 6 | 1.278(0.463~3.528) | 0.245(0.518) | 0.636 | 0.642(0.000) |  |  |  |
|  |  | MR-Egger | 6 | 0.909(0.033~5.372) | -0.096(1.699) | 0.958 |  | 0.053(0.843) |  |  |
|  |  | WM | 6 | 0.994(0.264~3.744) | -0.006(0.677) | 0.993 |  |  |  |  |
|  |  | PWM | 6 | 0.994(0.258~3.823) | -0.006(0.687) | 0.993 |  |  |  |  |
|  |  | MR.RAPS | 6 | 0.942(0.517~1.716) | -0.060(0.306) | 0.846 |  |  |  |  |
|  |  | CAUSE | 986 | 0.932(0.577~1.507) | 0.750(0.420) | 0.960 |  |  |  |  |
|  |  | MR-PRESSO | 6 | - | 0.025(0.366) | 0.949 |  |  | 8.873 | 0.468 |
| VEGF | Malignant Meninges | IVW | 6 | 0.831(0.486~1.421) | -0.185(0.273) | 0.499 | 0.083(0.487) |  |  |  |
|  |  | MR-Egger | 6 | 0.360(0.062~2.083) | -1.021(0.895) | 0.318 |  | 0.130(0.382) |  |  |
|  |  | WM | 6 | 0.610(0.354~1.049) | -0.495(0.277) | 0.075 |  |  |  |  |
|  |  | PWM | 6 | 0.592(0.351~1.000) | -0.523(0.267) | 0.050 |  |  |  |  |
|  |  | MR.RAPS | 6 | 1.309(1.041~1.644) | 0.269(0.116) | 0.021 |  |  |  |  |
|  |  | CAUSE | 986 | 1.073(0.878~1.323) | 0.760(0.970) | 0.780 |  |  |  |  |
|  |  | MR-PRESSO | 6 | - | -0.062(0.237) | 0.805 |  |  | 17.077 | 0.137 |

SNP: single-nucleotide polymorphism; SE: standard error; VEGF: vascular endothelial growth factor; IVW: inverse-variance weighted; WM: weighted median; PWM: penalty weighted median; MR.RAPS: robust adjusted profile score; CAUSE: causal analysis using summary effect estimates; MR-PRESSO: pleiotropy residual sum and outlier; OR: odds ratio; MR: Mendelian randomization; Q_pval: *P* value of the Cochran Q statistic; *I^2^* = (Q-df)/Q×100%; *P* < 0.05 were considered statistically significant.

**Supplementary Table 4** The results of CAUSE analyses

| model 1 | model 2 | delta_elpd | se _delta_elpd | | z | | *P* | delta_elpd | se _delta_elpd | | z | | *P* |
| --- | --- | --- | --- | --- | --- | --- | --- | --- | --- | --- | --- | --- | --- |
|  |  | VEGF to malignant brain tumor | | | | | | Malignant brain tumor to VEGF | | | | | |
| null | sharing | 0.27 | | 0.26 | | 1.10 | 0.86 | 0.12 | | 0.04 | | 2.90 | 1.00 |
| null | causal | 0.80 | | 0.76 | | 1.10 | 0.85 | 0.76 | | 0.20 | | 3.80 | 1.00 |
| sharing | causal | 0.53 | | 0.73 | | 0.73 | 0.77 | 0.64 | | 0.16 | | 3.90 | 1.00 |
|  |  | VEGF to brain glioblastoma | | | | | | Brain glioblastoma to VEGF | | | | | |
| null | sharing | 0.45 | | 0.12 | | 3.80 | 1.00 | 0.00 | | 0.01 | | 0.70 | 0.76 |
| null | causal | 1.20 | | 0.49 | | 2.40 | 0.99 | 0.07 | | 0.09 | | 0.74 | 0.77 |
| sharing | causal | 0.75 | | 0.42 | | 1.80 | 0.96 | 0.06 | | 0.09 | | 0.74 | 0.77 |
|  |  | VEGF to meninges | | | | | | Meninges to VEGF | | | | | |
| null | sharing | 0.14 | | 0.22 | | 0.64 | 0.74 | -0.01 | | 0.18 | | -0.05 | 0.48 |
| null | causal | 0.90 | | 1.10 | | 0.80 | 0.79 | -0.65 | | 1.50 | | -0.42 | 0.34 |
| sharing | causal | 0.76 | | 0.97 | | 0.78 | 0.78 | -0.64 | | 1.40 | | -0.46 | 0.32 |

CAUSE: causal analysis using summary effect estimates; VEGF: vascular endothelial growth factor.

**Supplementary Table 5** Causal associations of different types of malignant brain tumors with VEGF by reverse MR analyses

| Exposure | Outcome | Methods | nSNPs | β (95%CI) | *P* | Q_pval(*I^2^*) | Intercept(*P*) | RSSobs | Global *P* |
| --- | --- | --- | --- | --- | --- | --- | --- | --- | --- |
| Malignant brain tumor | VEGF | IVW | 12 | 0.005(-0.029~0.038) | 0.790 | 0.285(0.162) |  |  |  |
|  |  | MR-Egger | 12 | -0.010(-0.082~0.061) | 0.783 |  | 0.009(0.652) |  |  |
|  |  | WM | 12 | -0.003(-0.046~0.039) | 0.894 |  |  |  |  |
|  |  | PWM | 12 | -0.005(-0.048~0.039) | 0.839 |  |  |  |  |
|  |  | MR.RAPS | 12 | -0.009(-0.036~0.018) | 0.513 |  |  |  |  |
|  |  | CAUSE | 896 | 0.000(-0.120~0.110) | 1.000 |  |  |  |  |
|  |  | MR-PRESSO | 12 | -0.010(-) | 0.467 |  |  | 18.888 | 0.349 |
| Brain glioblastoma | VEGF | IVW | 13 | 0.010(-0.002~0.022) | 0.108 | 0.612(0.000) |  |  |  |
|  |  | MR-Egger | 13 | 0.016(-0.010~0.042) | 0.242 |  | -0.008(0.590) |  |  |
|  |  | WM | 13 | 0.012(-0.005~0.028) | 0.158 |  |  |  |  |
|  |  | PWM | 13 | 0.012(-0.005~0.029) | 0.179 |  |  |  |  |
|  |  | MR.RAPS | 13 | -0.002(-0.013~0.009) | 0.717 |  |  |  |  |
|  |  | CAUSE | 826 | 0.000(-0.110~0.110) | 0.770 |  |  |  |  |
|  |  | MR-PRESSO | 13 | -0.004(-) | 0.505 |  |  | 14.142 | 0.470 |
| Malignant meninges | VEGF | IVW | 12 | 0.010(-0.030~0.050) | 0.618 | 0.189(0.259) |  |  |  |
|  |  | MR-Egger | 12 | 0.046(-0.037~0.129) | 0.306 |  | -0.017(0.360) |  |  |
|  |  | WM | 12 | 0.008(-0.041~0.057) | 0.737 |  |  |  |  |
|  |  | PWM | 12 | 0.011(-0.039~0.060) | 0.670 |  |  |  |  |
|  |  | MR.RAPS | 12 | -0.004(-0.014~0.006) | 0.449 |  |  |  |  |
|  |  | CAUSE | 894 | 0.040(-0.010~0.090) | 0.320 |  |  |  |  |
|  |  | MR-PRESSO | 12 | 0.003(-) | 0.884 |  |  | 18.531 | 0.186 |

SNP: single-nucleotide polymorphism; VEGF: vascular endothelial growth factor; IVW: inverse-variance weighted; WM: weighted median; PWM: penalty weighted median; MR.RAPS: robust adjusted profile score; CAUSE: causal analysis using summary effect estimates; MR-PRESSO: pleiotropy residual sum and outlier; MR: Mendelian randomization; Q_pval: *P* value of the Cochran Q statistic; *I^2^* = (Q-df)/Q×100%; *P* < 0.05 was considered statistically significant.

1. **Supplementary Figures**


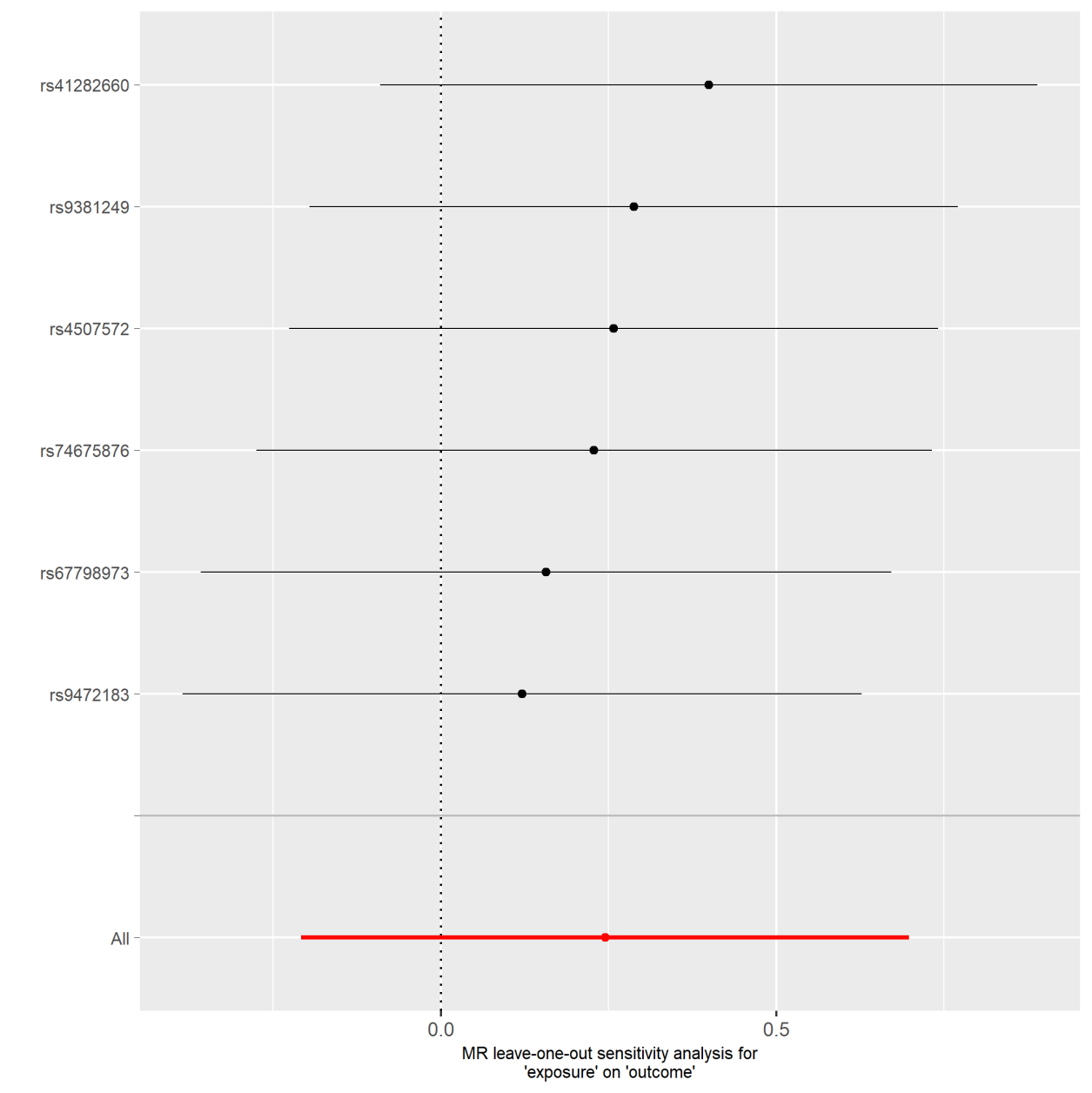


**Supplementary Figure 1** MR leave-one-out plot (VEGF for malignant brain tumor).

VEGF: vascular endothelial growth factor; MR: Mendelian randomization.


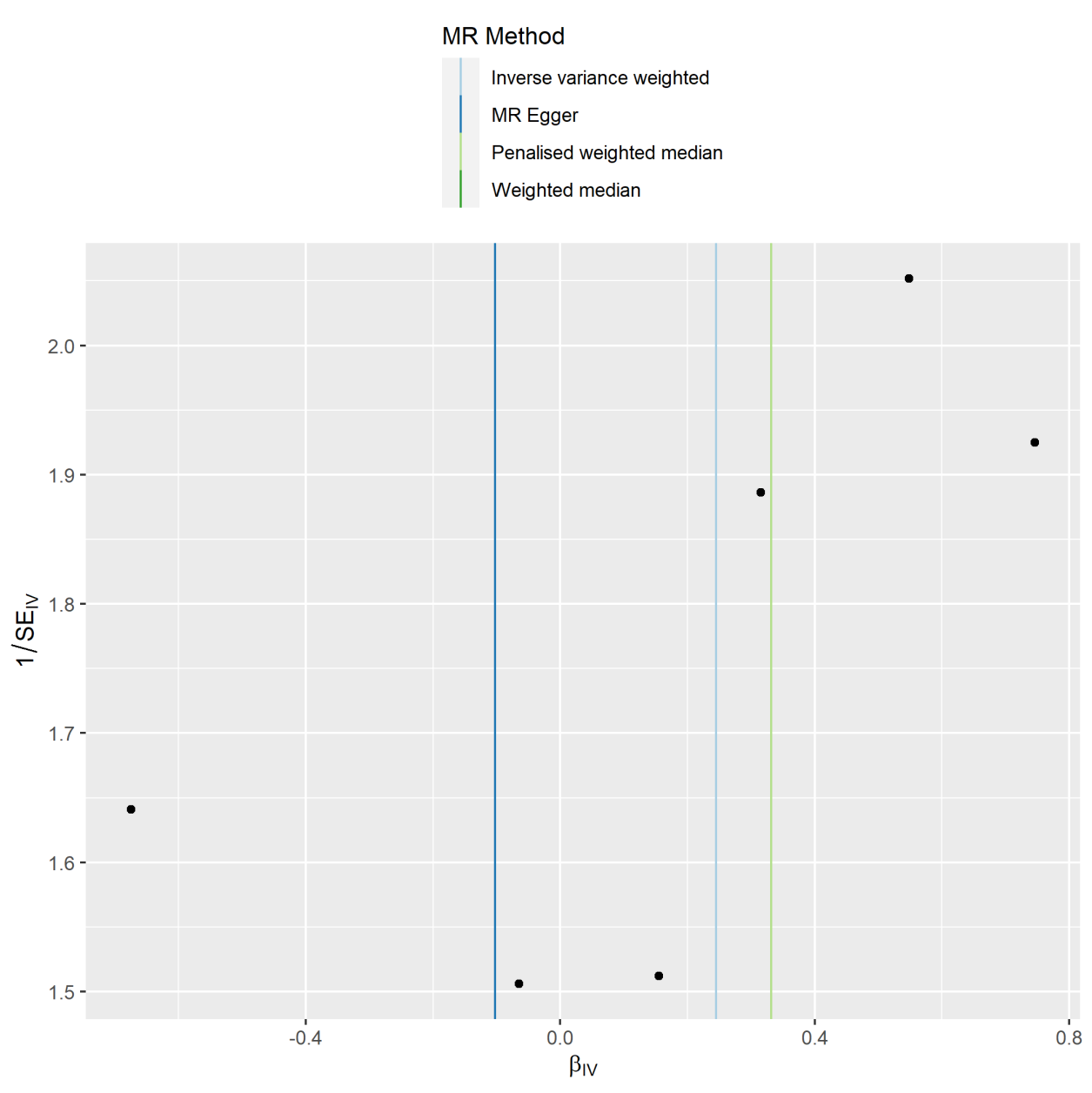


**Supplementary Figure 2** MR Funnel plot (VEGF for malignant brain tumor).

VEGF: vascular endothelial growth factor; MR: Mendelian randomization.


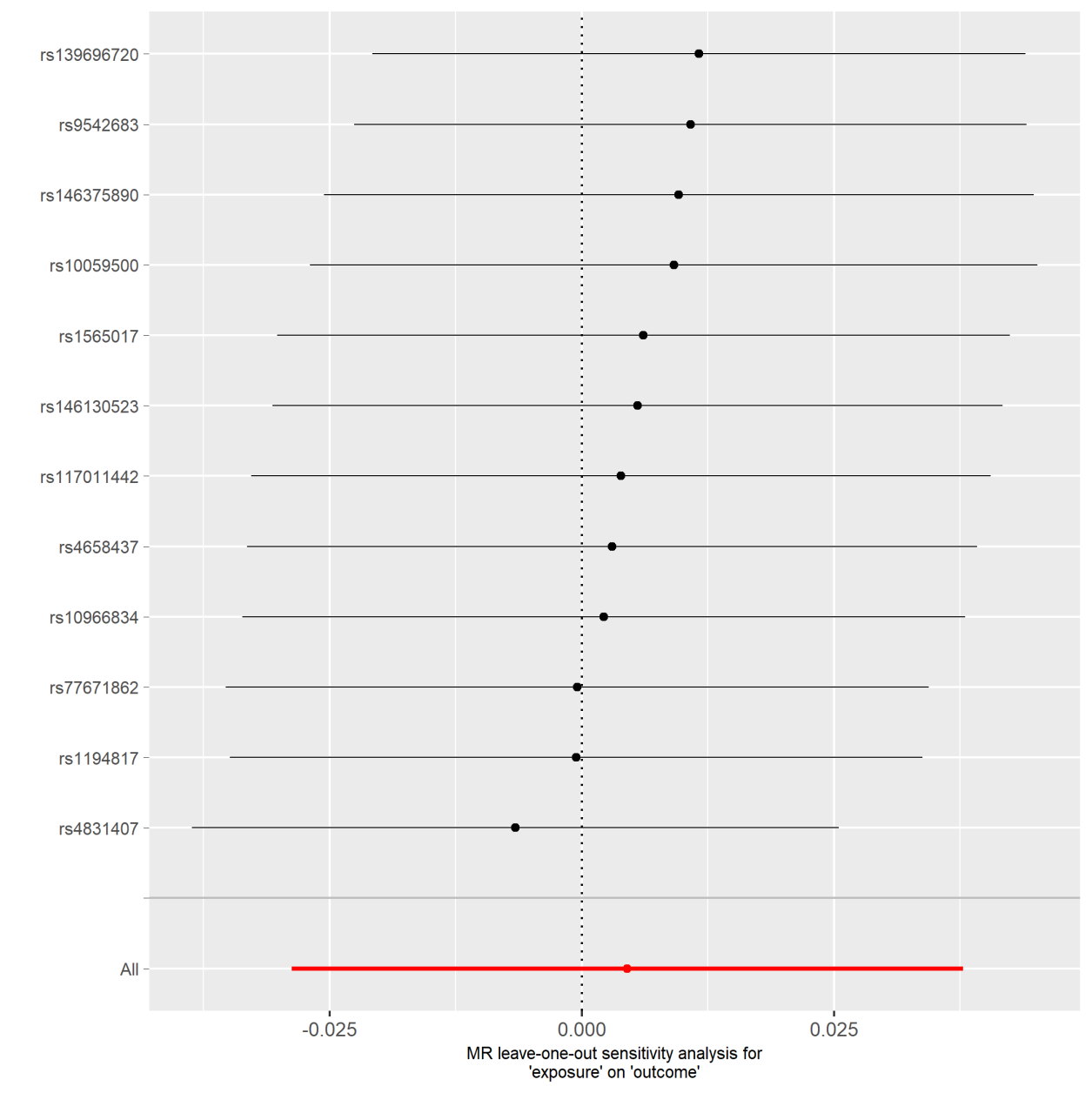


**Supplementary Figure 3** MR leave-one-out plot (malignant brain tumor for VEGF).

VEGF: vascular endothelial growth factor; MR: Mendelian randomization.


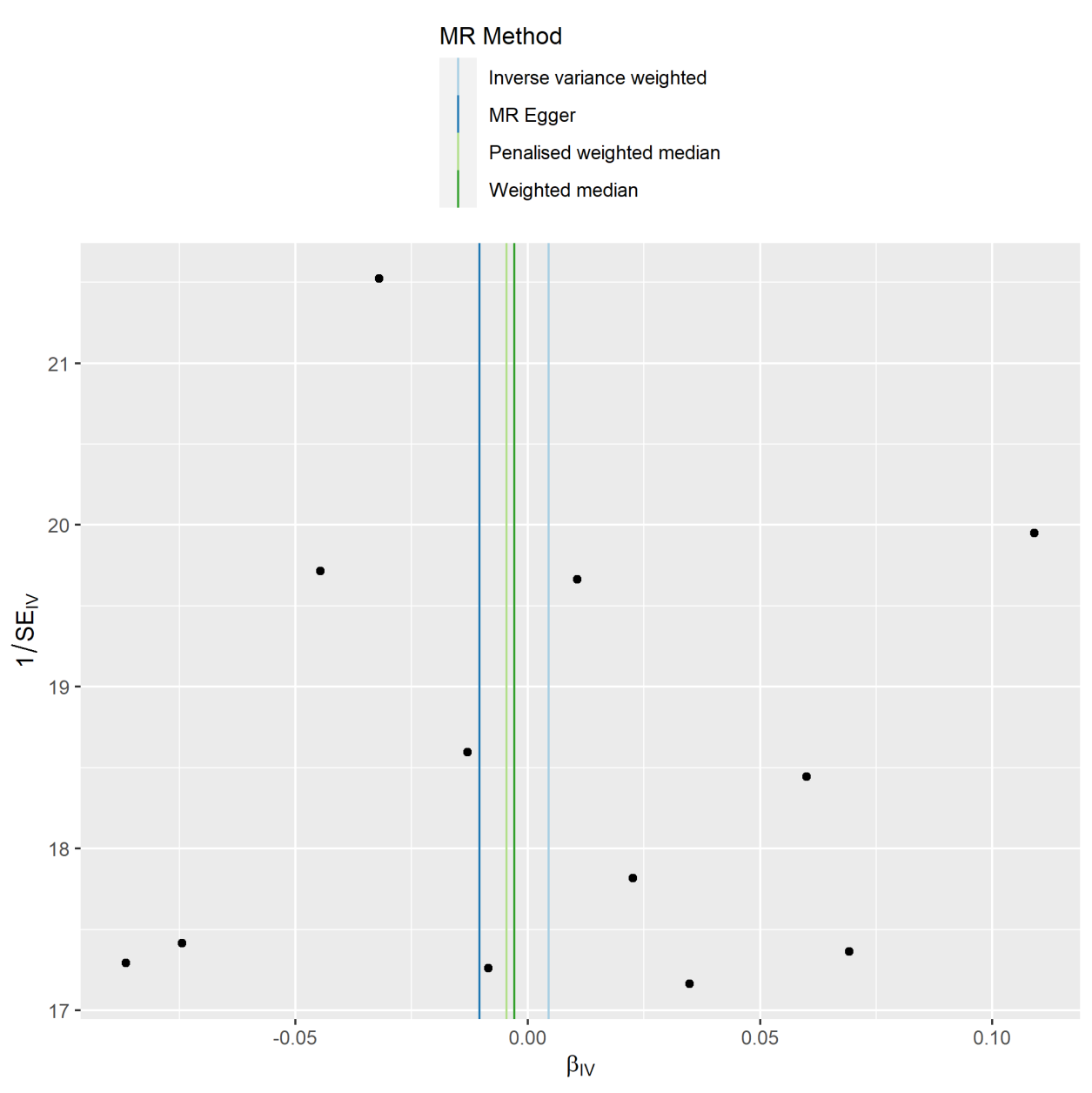


**Supplementary Figure 4** MR Funnel plot (malignant brain tumor for VEGF).

VEGF: vascular endothelial growth factor; MR: Mendelian randomization.
